# Understanding Motivation to Adhere to Guidelines for Alcohol Intake, Physical Activity, and Fruit and Vegetable Intake Among U.K. University Students

**DOI:** 10.1177/1090198120988251

**Published:** 2021-02-16

**Authors:** Richard O. de Visser, Dominic Conroy, Emma Davies, Richard Cooke

**Affiliations:** 1 University of Sussex, Falmer, UK; 2 University of East London, London, UK; 3 Oxford Brookes University, Oxford, UK; 4 University of Liverpool, Liverpool, UK

**Keywords:** alcohol, diet, guidelines, motivation, physical activity

## Abstract

**Background:**

To encourage people to lead healthier lifestyles, governments in many countries publish guidelines for alcohol intake, physical activity (PA), and fruit and vegetable (FV) intake. However, there is a need for better understanding of whether people understand such guidelines, consider them useful, and adhere to them. University students are a group worthy of attention because although they are less likely than older adults to exceed U.K. weekly alcohol intake guidelines or to be inactive, they are also less likely to meet FV consumption targets. Furthermore, because behavior during youth predicts adult behavior, it is important to identify influences on healthier behavior.

**Method:**

An online survey was completed by 559 U.K. university students. Key outcome variables were knowledge of guidelines, motivation to adhere to them, and adherence to them.

**Results:**

A total of 72% adhered to guidelines for alcohol intake, 58% for PA, and 20% for FV intake. Students generally had poor or moderate knowledge of guidelines, perceived them as only moderately useful, and were only moderately motivated to adhere to them. Greater motivation to adhere to guidelines was not significantly related to more accurate knowledge. However, it was related to greater familiarity, and perceiving guidelines as useful and realistic, and greater conscientiousness.

**Discussion:**

There is a need to ensure that students understand the U.K. guidelines for alcohol intake, PA, and FV intake. However, simply increasing knowledge may not lead to greater adherence to the guidelines: There is also a need to focus on improving perceptions of how useful and realistic they are.

In its global action plan for noncommunicable diseases, the World Health Organization (WHO, 2013) specified nine global behavior targets. One was to reduce the harmful use of alcohol by at least 10%. Another was to reduce the prevalence of insufficient physical activity (PA) by at least 10%. There was no specific target for fruit and vegetable (FV) intake, but diet was relevant to cardiovascular diseases, cancer, diabetes, and obesity targets. Guidelines for healthy lifestyles are important counterparts to such targets, and the WHO (2020) considers the development of evidence-based global guidelines to be one of its core functions. Guidelines are required to specify recommended behavior using clearly defined and shared terms that allow members of the public, health professionals, and researchers to “speak the same language” so that behavior change can be measured objectively.

Although the WHO (2010b) has no global guidelines for alcohol intake, many countries have developed these (Furtwängler & de Visser, 2013). In the United Kingdom, adults are advised to drink no more than 14 units (total 112 g) of alcohol per week, and to have at least two alcohol-free days per week (U.K. Department of Health, 2016). The WHO (2010a) advises adults to engage in at least 150 minutes of moderate activity or at least 75 minutes of vigorous activity per week. This is reflected in the U.K. guidelines, which also advise regular muscle strengthening activities (Chief Medical Officers [CMO], 2019). In relation to diet, the WHO (2013) recommends that adults consume at least five servings (total 400 g) of fruit and vegetables per day, consume less than 5 g of salt or 2 g of sodium per day, and limit their intake of saturated fats. U.K. guidelines make the same recommendations for FV intake, and similar recommendations for other elements of diet (Public Health England [PHE], 2016, 2018).

Many people fail to meet these guidelines: In the 2018 Health Survey for England, 30% of men and 14% of women exceeded the weekly alcohol intake guideline, 26% of men and 27% of women were physically inactive, and 75% of men and 70% of women did not eat five or more FV servings per day (NHS Digital, 2019). However, the definition of “inactivity” used in the Health Survey for England—that is, less than 30 minutes of PA per week—does not map onto the CMO (2019) guidelines for PA. A summary of research involving over 1.9 million people worldwide found that 28% were insufficiently physically active, and that in high-income countries, women were less likely than men to meet PA targets (Guthold et al., 2018).

Accurate knowledge of guidelines is one focus for interventions. However, enduring behavior change is unlikely to arise simply from increasing knowledge of guidelines. The information-motivation-behavioral skills model argues that in addition to possessing accurate information, people must be motivated to change, and must have the behavioral skills required to carry out new patterns of behavior (Fisher et al., 2003). In addition, the COM-B (capability, opportunity, motivation, behavior) model (Michie et al., 2014) emphasizes the importance of capability (i.e., skills and confidence) to carry out new behaviors, opportunities to develop and implement this capability, and motivation to change. In the context of alcohol use, intervention research provides support for these models (de Visser et al., 2017; de Visser & Piper, 2020). Although there are no peer-reviewed evaluations of their impact, campaigns such as “Meat-Free Mondays” (www.meatfreemondays.com) and “Move for Movember” (uk.movember.com/get-involved/move) provide structured and supported opportunities for people to change their behavior. Similarly, stop-smoking campaigns such as “Stoptober” provide a structured and supported opportunity for smokers to initiate and maintain behavior change (Brown et al., 2014). Information may be important to encourage contemplation of behavior change, but motivation and skills are required for initiating and maintaining behavior change (Prochaska & DiClemente, 1984).

There is not a simple link from knowledge of guidelines to adherence to them (de Visser & Birch, 2012; Roth & Stamatakis, 2010). Studies of unit-based guidelines for alcohol intake indicate that people often lack confidence in applying them to their own behavior (de Visser, 2015; de Visser & Birch, 2012; Furtwängler & de Visser, 2017a, 2017b). Furthermore, many people have negative attitudes toward the guidelines, and low motivation to adhere to them (Burgess et al., 2019; Furtwängler & de Visser, 2017a, 2017b). One reason that people give for not intending to adhere to alcohol guidelines is that they do not consider them to be realistic—hereafter “perceived realism” (Furtwängler & de Visser, 2017a, 2017b; Lovatt et al., 2015); there is a lack of equivalent research for PA and FV intake guidelines. Theories of communication emphasize the importance of the perceived trustworthiness and credibility of sources of advice (Cairns et al., 2013; Eagly & Chaiken, 1993; Pornpitakpan, 2004; Sbaffi & Rowley, 2017), but scant attention has been given to whether message recipients consider advice to be realistic and applicable to them. Furthermore, a key feature of “SMART” goals is that they are perceived by individuals to be realistic (Doran, 1981; Shaw et al., 2015). It is therefore important to explore audience beliefs about message content, not just the credibility and trustworthiness of the message source.

In addition to specific knowledge and attitudes, it is important to acknowledge the influence of more general characteristics of individuals, including personality dimensions. For example, more conscientious people are less likely to engage in risky behaviors, more likely to engage in healthy behaviors, and have longer life expectancy (Bogg & Roberts, 2004, 2013). Among young people, conscientiousness is related to healthier patterns of alcohol use (Hagger-Johnson et al., 2012), physical activity (Kroencke et al., 2019), and diet (Conner et al., 2017).

The aim of the study reported here was to explore associations between knowledge of, beliefs about, and adherence to U.K. government guidelines for alcohol intake, PA, and FV intake. Particular attention was given to motivation to adhere to the different guidelines. A sample of university students was chosen because although young people are less likely than older adults to drink excessively or to be inactive, they are also less likely to meet FV consumption target (NHS Digital, 2019). Such differences in behavior may reflect different patterns of knowledge, beliefs, and behavior in different population segments, and may suggest a need to develop tailored policies and interventions for different segments of the population (such as university students). Furthermore, behavior during adolescence and young adulthood predicts adult behavior (Daw et al., 2017; Salin et al., 2019), so it is important to identify and address determinants of healthier behavior and to use this information to identify effective ways to encourage healthier behavior.

## Method

### Sample

Questionnaires were completed by 559 U.K. university students (424 women, 133 men, 2 other) with a mean age of 22.8 years (*SD* = 8.05). The completion rate was 73%: 206 incomplete questionnaires were excluded from analysis. The ethnic profile of the sample was comparable to that for U.K. universities in general: 77% White, 10% Asian, 7% Black, 3% mixed ethnicity, 3% other (HM Government, 2020).

Email invitations to participate in the study were sent on behalf of the researchers by administrative staff at four universities. Participants could enter a draw for two £25 prizes or receive research participation credits (for psychology students at some universities). Ethical approval was granted by each institution. Because the number of eligible people (currently enrolled students aged 18–30 years) who received the invitation is unknown, the response rate cannot be calculated.

### Measures

In addition to providing demographic data, respondents used 5-point scales to rate their physical health, sleep quality, energy levels, and concentration (de Visser & Piper, 2020).

#### Knowledge of Guidelines

Fourteen items assessed knowledge of alcohol intake guidelines. The first two assessed whether participants knew the recommended maximum weekly unit intake for women, and for men (de Visser et al., 2017). Two novel items assessed knowledge of the recommended number of alcohol-free days per week for women, and for men. Knowledge of the unit content of different drinks was assessed with 10 items (de Visser et al., 2017). Color pictures of beer, wine, and spirits were accompanied by brief descriptions: for example, “pint (568 mL) of regular strength beer.” Estimates were typed into a free-text box, and were considered to be correct if within ±10% of the actual unit content. Correct responses to the 14 items were summed and divided by 1.4 to give knowledge scores from 0 to 10 comparable to those for other behaviors. The alcohol knowledge measures taken from de Visser et al. (2017) were adapted to assess knowledge of PA guidelines and FV guidelines.

The first PA item assessed knowledge of the recommended minimum weekly amount of moderate activity or vigorous activity: Respondents provided answers in a free-text box. Knowledge of the PA level of different behaviors was assessed with 10 items. Respondents indicated whether they thought that the behaviors would be considered “vigorous,” “moderate,” or “neither.” The list of 10 behaviors contained four vigorous activities (e.g., “Aerobics/Zumba/etc.”), three moderate activities (e.g., “Pushing a lawnmower”), and three other activities (e.g., “washing a car”). Correct responses to the 11 items were summed and divided by 1.1 to give knowledge scores from 0 to 10 comparable to those for other behaviors.

The first FV item assessed knowledge of the recommended minimum daily number of servings of fruit and vegetables that an adult should eat: Respondents provided answers in a free-text box. The next item assessed knowledge of the mass of one serving of FV using a drop-down menu (20 g, 40 g, 60 g, . . ., 200 g). Knowledge of the number of servings of FV in different food portions was assessed with 10 items. Respondents used drop-down menus (0 servings, 0.5 servings, 1 serving, . . ., 5 servings) to indicate how many servings they thought were in portions such as “1 apple, 1 banana.” Correct responses to the 12 items were summed and divided by 1.2 to give knowledge scores from 0 to 10 comparable to other behaviors.

#### Perception of Guidelines and Motivation to Adhere

Respondents used scales ranging from 0 = *not at all certain* to 10 = *completely certain* to indicate how certain they were that they knew what was meant by “a unit of alcohol,” “moderate activity,” and “one serving of fruit/vegetables.”

Respondents used similar scales adapted from de Visser et al. (2017) ranging from *not at all* to 10 = *completely* to respond to the following five stem statements tailored to each of the three behavioral guidelines: “How familiar are you with . . .?”; “How useful to you is . . .?”; “How useful to you would it be to know more about . . .?”; “How realistic are the guidelines for . . .?”; and “How motivated are you to adhere to the guidelines for . . .?”

#### Behavior

Behavior was assessed with measures that allowed creation of three dichotomous variables indicating whether respondents adhered to guidelines for alcohol intake, PA, and FV intake.

Participants viewed a pictorial guide to the unit content of various drinks and used it to report how many units they consumed each day of the previous week (de Visser, 2015). Responses were used to compute the total number of units in the last week: Those who consumed 0 to 14 units were coded as adhering to the alcohol intake guidelines, those who consumed 15+ units were coded as not adhering.

Participants viewed a pictorial guide to “moderate” and “vigorous” PA and used it to report how many minutes of moderate PA and how many minutes of vigorous PA they engaged in during the previous week. If either response was above the recommended minimum, then respondents were coded as adhering to the PA guidelines, otherwise they were coded as not adhering.

Participants viewed a pictorial guide to the number of FV servings in various portions of food and used it to report how many FV servings they consumed each day of the previous week. Respondents who reported consuming at least five servings per day were coded as adhering to the FV guidelines, otherwise they were coded as not adhering.

Knowledge questions were presented before behavior questions. The questionnaire prohibited back-tracking: The guides used to aid reports of behavior could not be used to amend responses to knowledge questions.

### Analysis

Within-subject analyses of variance (ANOVAs) provided descriptive data and allowed comparisons of guideline-related knowledge, perceptions, motivation, and behavior across the three domains. Because of the repeated comparison across the three behaviors, the conventional significance level was reset at .017 (i.e., .05/3).

## Results

### Descriptive Data

Given the 10-point scales, we defined scores up to 3 to be *low*, 4 to 6 to be *moderate*, and scores 7 to 10 to be *high*. Across all three domains, respondents had low to moderate levels of knowledge, certainty in knowledge, and familiarity with guidelines (Table 1). They perceived moderate levels of utility and realism. There was not a perception that more information would be useful. Motivation to adhere to guidelines was moderate.

**Table 1.** Knowledge, Beliefs, and Adherence to Guidelines for Healthy Lifestyles (*n* = 559).

Variable	Alcohol intake, *M* (*SD*)	Physical activity, *M* (*SD*)	Fruit/vegetable intake, *M* (*SD*)
Knowledge	1.97 (1.43)^a^	5.01 (1.22)^b^	4.86 (1.82)^b^
Perceived certainty	4.18 (2.93)^a^	5.99 (2.62)^b^	3.60 (2.60)^a^
More information would be useful	4.98 (2.96)	6.16 (2.73)	6.04 (2.84)
Familiarity with guideline	4.81 (2.97)	4.54 (2.91)	5.60 (2.84)
Perceived utility of guideline	3.38 (3.00)	5.58 (2.70)	6.06 (2.68)
Perceived realism of guideline	4.61 (2.70)	6.52 (2.30)	6.34 (2.49)
Motivation to adhere to guideline	4.41 (3.46)	6.48 (2.70)	6.76 (2.61)
Adhered to guideline, %	72.3	57.8	20.4

*Note*. For continuous variables, range 0 to 10 different superscripts in rows indicate significantly different mean scores.

The majority of respondents adhered to the guidelines for alcohol intake and PA, but a minority adhered to the FV intake guidelines. Men were significantly more likely to meet the PA guidelines (χ^2^_(1)_ = 13.62, *p* < .01). There were no significant sex differences for alcohol intake (χ^2^_(1)_ = 2.40, *p* = .12) or FV intake (χ^2^_(1)_ = 0.09, *p* = .76). People of non-White ethnicity were significantly more likely to adhere to the alcohol guidelines (χ^2^_(1)_ = 7.50, *p* < .01), partially because they were less likely than White respondents to drink (49% vs. 77%). There were no significant ethnicity differences for PA (χ^2^_(1)_ = 1.96, *p* = .16) or FV intake (χ^2^_(1)_ = 0.44, *p* = .51). Subject of study was not significantly related to adherence to guidelines for alcohol (χ^2^_(1)_ = 0.33, *p* = .57), PA (χ^2^_(1)_ = 5.29, *p* = .02), or FV intake (χ^2^_(1)_ = 1.81, *p* = .18).

Within-subjects ANOVA revealed that knowledge of the alcohol guidelines was significantly lower than knowledge of the PA or FV guidelines (*F*_(2, 556)_ = 20.68, *p* < .01). Respondents had significantly greater certainty in their knowledge of the definition of moderate PA than they did for a unit of alcohol or a serving of FV (*F*_[2, 556]_ = 18.60, *p* < .01). However, across the three behavioral domains, there were comparable levels of familiarity (*F*_(2, 556)_ = 0.67, *p* = .51), perceived utility (*F*_(2, 556)_ = 2.23, *p* = .11), perceived realism (*F*_(2, 556)_ = 1.83, *p* = .16), and motivation to adhere to guidelines (*F*_(2, 556)_ = 0.53, *p* = .59).

### Correlations Between Cognitive Components

Correlations between motivation to adhere to the different guidelines were significant but not so strong as to suggest multicollinearity (alcohol–PA: *r* = .27, *p* < .01; alcohol–FV intake: *r* = .28, *p* < .01; PA–FV intake: *r* = .46, *p* < .01; variance inflation factor [VIF] = 1.28). Greater conscientiousness was related to stronger motivation to adhere to guidelines for alcohol intake and FV intake, but not PA (Table 2). Accuracy of knowledge of guidelines was not significantly related to motivation to adhere to them. However, for each guideline, greater motivation to adhere was related to greater familiarity with it, and perceiving that it was more useful and more realistic. The correlations generally indicated “medium” effect sizes: The realism–motivation associations for PA and FV intake were “large” (Cohen, 1988).

**Table 2.** Bivariate Correlations Among Beliefs About Guidelines (*n* = 559).

Guideline	Familiarity	Usefulness	Realism	Motivation
Alcohol intake				
Age	*r* = .14^a^ (*p* < .01)	*r* = .18^a^ (*p* ≤ .01)	*r* = .15^a^ (*p* ≤ .01)	*r* = .19^a^ (*p* ≤ .01)
Health	*r* = .10 (*p* = .02)	*r* = .12^a^ (*p* < .01)	*r* = .09 (*p* = .03)	*r* = .10 (*p* = .02)
Conscientiousness	*r* = .09 (*p* = .04)	*r* = .03 (*p* = .47)	*r* = .07 (*p* = .09)	*r* = .13^a^ (*p* < .01)
Knowledge	*r* = .30^b^ (*p* < .01)	*r* = .13^a^ (*p* < .01)	*r* = .18^a^ (*p* < .01)	*r* = .05 (*p* = .28)
Reported certainty	*r* = .63^c^ (*p* < .01)	*r* = .42^b^ (*p* < .01)	*r* = .25^a^ (*p* < .01)	*r* = .11^a^ (*p* < .01)
Familiarity		*r* = .46^b^ (*p* < .01)	*r* = .24^a^ (*p* < .01)	*r* = .11^a^ (*p* < .01)
Perceived usefulness			*r* = .39^b^ (*p* < .01)	*r* = .37^b^ (*p* < .01)
Perceived realism				*r* = .37^b^ (*p* < .01)
Physical activity				
Age	*r* = .04 (*p* = .34)	*r* = .06 (*p* = .14)	*r* = .09 (*p* = .03)	*r* = .06 (*p* = .18)
Health	*r* = .15^a^ (*p* < .01)	*r* = .13^a^ (*p* < .01)	*r* = .11^a^ (*p* = .01)	*r* = .23^a^ (*p* < .01)
Conscientiousness	*r* = .13^a^ (*p* < .01)	*r* = .11^a^ (*p* < .01)	*r* = .06 (*p* = .11)	*r* = .08 (*p* = .07)
Knowledge	*r* = .25^a^ (*p* < .01)	*r* = .15^a^ (*p* < .01)	*r* = .11 (*p* = .01)	*r* = .07 (*p* = .10)
Reported certainty	*r* = .34^b^ (*p* < .01)	*r* = .23^a^ (*p* < .01)	*r* = .19^a^ (*p* < .01)	*r* = .15^a^ (*p* < .01)
Familiarity		*r* = .42^b^ (*p* < .01)	*r* = .27^a^ (*p* < .01)	*r* = .30^b^ (*p* < .01)
Perceived usefulness			*r* = .38^b^ (*p* < .01)	*r* = .51^c^ (*p* < .01)
Perceived realism				*r* = .47^b^ (*p* < .01)
Fruit/vegetable intake				
Age	*r* = .09 (*p* = .04)	*r* = .11 (*p* < .01)	*r* = .13 (*p* < .01)	*r* = .13 (*p* < .01)
Health	*r* = .16^a^ (*p* < .01)	*r* = .11^a^ (*p* < .01)	*r* = .20^a^ (*p* < .01)	*r* = .15^a^ (*p* < .01)
Conscientiousness	*r* = .15^a^ (*p* < .01)	*r* = .12^a^ (*p* < .01)	*r* = .16^a^ (*p* < .01)	*r* = .18^a^ (*p* < .01)
Knowledge	*r* = .11^a^ (*p* = .01)	*r* = .07 (*p* = .10)	*r* = .08 (*p* = .06)	*r* = .07 (*p* = .11)
Reported certainty	*r* = .40^b^ (*p* < .01)	*r* = .27^a^ (*p* < .01)	*r* = .17^a^ (*p* < .01)	*r* = .15^a^ (*p* < .01)
Familiarity		*r* = .45^b^ (*p* < .01)	*r* = .30^b^ (*p* < .01)	*r* = .31^b^ (*p* < .01)
Perceived usefulness			*r* = .41^b^ (*p* < .01)	*r* = .33^b^ (*p* < .01)
Perceived realism				*r* = .52^c^ (*p* < .01)

a Small effect. ^b^Medium effect. ^c^Large effect (Cohen, 1988).

### Correlates of Motivation to Adhere to Guidelines

There were no significant sex differences in motivation to adhere to the guidelines for alcohol (*F*_(1,557)_ = 0.86, *p* = .35), PA (*F*_(1,557)_ = 0.36, *p* = .55), or FV intake (*F*_(1,557)_ = 5.62, *p* = .02). There were no significant ethnicity differences in motivation for alcohol (*F*_(1,557)_ = 0.08, *p* = .78), PA (*F*_(1,557)_ = 0.05, *p* = .82), or FV intake (*F*_(1,557)_ = 0.08, *p* = .78). Subject of study was not significantly related to motivation to adhere to guidelines for alcohol (*F*_(1, 464)_ = 2.51, *p* = .11), PA (*F*_(1, 464)_ = 0.24, *p* = .63), or FV intake (*F*_(1, 464)_ = 0.07, *p* = .80).

Greater motivation to adhere to the guidelines was expressed by respondents who already adhered to them (alcohol: 4.99 vs. 2.89; *F*_(1,557)_ = 48.08, *p* < .01; PA: 7.82 vs. 6.50; *F*_(1,557)_ = 26.41, *p* < .01; FV: 6.96 vs. 5.83; *F*_(1,557)_ = 27.58, *p* < .01). These results and those in Table 2 informed regression analyses: Significant bivariate correlates of motivation were entered into a “forward selection” stepwise process of model building. Table 3 displays the final models for each guideline.

**Table 3.** Multivariate Correlates of Motivation to Adhere to Guidelines (*n* = 559).

Guideline	Unstandardized Β (*SE*)	β	*T*
Alcohol intake			
*F*_(3, 553)_ = 82.75, *p* < .01			
Adjusted *R*^2^ = .31			
Perceived realism	0.44 (0.05)	.34	8.88, *p* < .01
Met guidelines?	1.62 (0.27)	.23	6.11, *p* < .01
Perceived usefulness	0.27 (0.04)	.22	6.10, *p* < .01
Physical activity			
*F*_(4,552)_ = 90.05, *p* < .01			
Adjusted *R*^2^ = .39			
Perceived usefulness	0.39 (0.04)	.38	10.66, *p* < .01
Perceived realism	0.34 (0.04)	.29	8.14, *p* < .01
Met guidelines?	0.72 (0.18)	.14	4.07, *p* < .01
Physical health	0.37 (0.10)	.13	3.87, *p* < .01
Fruit/vegetable intake			
*F*_(4, 552)_ = 78.48, *p* < .01			
Adjusted *R*^2^ = .36			
Perceived realism	0.42 (0.04)	.40	10.88, *p* < .01
Perceived usefulness	0.26 (0.04)	.27	7.34, *p* < .01
Met guidelines?	0.84 (0.22)	.13	3.88, *p* < .01
Physical health	0.26 (0.09)	.10	2.72, *p* < .01

Greater motivation to adhere to alcohol guidelines was significantly related to greater perceived realism of the guidelines, currently meeting the guidelines, and perceiving the guidelines to be more useful. Greater motivation for PA guidelines was significantly related to perceiving the guidelines to be more useful, perceiving them to be more realistic, currently meeting the guidelines, and reporting better health. Greater motivation for FV guidelines was significantly related to perceiving the guidelines to be more realistic, perceiving them to be more useful, currently meeting the guidelines, and reporting better health.

## Discussion

Our findings do not paint a positive picture of U.K. university students’ knowledge, attitudes, and practices related to guidelines for healthy living. Across the three behavioral domains of alcohol intake, physical activity, and fruit and vegetable intake, respondents lacked accurate knowledge of the guidelines, and did not feel familiar with them. Furthermore, they tended not to perceive the guidelines to be useful or realistic, and they were not highly motivated to adhere to them. Although 73% adhered to the weekly intake guidelines for alcohol use, only 58% adhered to the PA guidelines, and 20% adhered to the FV guidelines. These figures are notably different from the respective figures of approximately 20%, 25%, and 70% found among the general population (NHS Digital, 2019). Such differences may have implications for policy and practice: They highlight a need to identify patterns of knowledge, beliefs, and behavior in different population segments, and to develop tailored policies and interventions where appropriate. Effective policies and interventions could help to establish healthy patterns of behavior early in adult life (Daw et al., 2017; Salin et al., 2019).

Perceived familiarity with guidelines was not a consistent correlate of actual knowledge. Furthermore, respondents expressed low levels of certainty of knowing what is meant by “a unit” of alcohol, “moderate/vigorous” PA, or a “serving” of FV. These findings suggest a need to address accuracy of knowledge of guidelines by providing definitions that are easier to understand. However, respondents tended not to express strong beliefs that more information about guidelines would be useful, and accuracy of knowledge of guidelines was not significantly related to motivation to adhere to them. As noted elsewhere, information per se may not be sufficient to change behavior: Motivation and skills are also important (Brown et al., 2014; de Visser et al., 2017; de Visser & Birch, 2012; de Visser & Piper, 2020; Fisher et al., 2003; Michie et al., 2014; Prochaska & DiClimente, 1984; Roth & Stamatakis, 2010). Other studies have had some success in using personal feedback to promote healthier beliefs, motivation, and behavior (e.g., de Visser, 2015), and there is increasing evidence of the value of using mobile technologies to deliver ecological momentary interventions to motivate and monitor healthier behavior (Heron & Smyth, 2010; Villinger et al., 2019)

Multivariate analyses identified very similar predictors of stronger motivation to adhere to guidelines for all three behaviors: Greater perceived realism of the guidelines, greater perceived utility of the guidelines, and already meeting the guidelines. In addition, better physical health was associated with stronger motivation to adhere to the PA and FV intake guidelines, suggesting a need to help people with poorer health to become more motivated to adhere. The results also suggest a need for further studies of how to improve the perceived realism and perceived utility of guidelines. As noted in the introduction, although sending messages from a credible and trustworthy source such as the CMO is important, so too are audience beliefs about the realism and applicability of message content (Cairns et al., 2013; Doran, 1981; Eagly & Chaiken, 1993; Pornpitakpan, 2004; Sbaffi & Rowley, 2017). It is important to explore further the impact of perceived realism, given that the impact of messages is affected by the target audience’s perceptions of whether they are personally relevant, and whether they would be effective (Furtwängler & de Visser, 2017a, 2017b; Noar et al., 2020).

Research including a qualitative component could help to identify barriers to adherence to guidelines, including ideas for how to make it easier for people to understand terms like “units,” “portions,” “moderate,” and “vigorous,” and how to apply this knowledge to their own behavior. For example, many people find alcohol units abstract and difficult to add up (Furtwängler & de Visser, 2017b). Future work could explore how technology could best be used to enhance knowledge and facilitate behavior change (Palmer et al., 2018)

### Limitations and Future Directions

This study was the first to explore students’ understanding and use of government guidelines for healthy lifestyles in multiple domains. However, it had some limitations. There is a need to expand the focus beyond U.K. university students to include more demographically diverse groups in more countries, because the health behaviors of university students may not be comparable to those other young people or older adults (de Visser et al., 2005). Although the ethnic profile of the sample was similar to that of the broader body of U.K. university students (HM Government, 2020), there was a preponderance of female respondents. Although results were not related to ethnicity or subject of study, in at least some domains there is cross-cultural variation in guidelines for healthy lifestyles (Furtwängler & de Visser, 2013). However, research in the domain of alcohol suggests that similar processes operate among students and nonstudent adults (de Visser, 2015; de Visser & Birch, 2012; de Visser et al., 2017). Furthermore, the study relied on self-reports of behavior, with no external validation of these: But it should also be noted that self-reports are commonly used in studies similar to ours, and in government-funded population-level research (e.g., NHS Digital, 2019).

A primary focus of this cross-sectional survey was motivation to adhere to guidelines. Future research could focus on whether people have requisite skills to enact behavior change (Fisher et al., 2003; Michie et al., 2014), ideally using prospective longitudinal designs. It may be possible to apply methods used in studies of alcohol use (de Visser, 2015; de Visser et al., 2017) to the domains of PA and FV intake to enhance knowledge of guidelines, and motivation and skills required to use them. Future research could also broaden the behavioral focus in each domain. This study focused on a key behavior in each domain to reduce questionnaire length. In relation to PA, future work could also focus on engagement in muscle strengthening activities (CMO, 2019; WHO, 2010a). In the dietary domain, future work could also focus on fat and salt intake (PHE, 2016, 2018; WHO, 2013).

### Conclusion

This study of university students was the first to assess engagement with government guidelines across multiple behavioral domains. It revealed that students tended to have poor knowledge of healthy lifestyle guidelines, only considered them moderately useful, and were not strongly motivated to adhere to them. The analyses also indicated that increasing people’s understanding of healthy lifestyle guidelines may not lead to greater motivation to adhere to them: There is also a need to focus on improving perceptions of how useful and realistic they are.
